# Subaqueous 3D stem cell spheroid levitation culture using anti-gravity bioreactor based on sound wave superposition

**DOI:** 10.1186/s40824-023-00383-w

**Published:** 2023-05-19

**Authors:** Jung Hwan Park, Ju-Ro Lee, Sungkwon Park, Yu-Jin Kim, Jeong-Kee Yoon, Hyun Su Park, Jiyu Hyun, Yoon Ki Joung, Tae Il Lee, Suk Ho Bhang

**Affiliations:** 1 School of Chemical Engineering, Sungkyunkwan University, Suwon, 16419 Republic of Korea; 2 Center for Biomaterials, Biomedical Research Institute, Korea Institute of Science and Technology, Seoul, 02792 Republic of Korea; 3 Department of Food Science and Biotechnology, College of Life Science, Sejong University, Seoul, 05006 Korea; 4 Department of Systems Biotechnology, Chung-Ang University, Gyeonggi-Do, Anseong-Si, 17540 Republic of Korea; 5 Division of Bio-Medical Science and Technology, University of Science and Technology, Republic of Korea, Seoul, 02792 Republic of Korea; 6 Department of Materials Science and Engineering, Gachon University, Gyeonggi-Do, Seongnam-Si, 13120 Republic of Korea

**Keywords:** Acoustic levitation, Anti-gravity bioreactor, Human mesenchymal stem cell spheroid, Hindlimb ischemia

## Abstract

**Background:**

Recently, various studies have revealed that 3D cell spheroids have several advantages over 2D cells in stem cell culture. However, conventional 3D spheroid culture methods have some disadvantages and limitations such as time required for spheroid formation and complexity of the experimental process. Here, we used acoustic levitation as cell culture platform to overcome the limitation of conventional 3D culture methods.

**Methods:**

In our anti-gravity bioreactor, continuous standing sonic waves created pressure field for 3D culture of human mesenchymal stem cells (hMSCs). hMSCs were trapped and aggerated in pressure field and consequently formed spheroids. The structure, viability, gene and protein expression of spheroids formed in the anti-gravity bioreactor were analyzed by electron microscope, immunostaining, polymerase chain reaction, and western blot. We injected hMSC spheroids fabricated by anti-gravity bioreactor into the mouse hindlimb ischemia model. Limb salvage was quantified to evaluate therapeutic efficacy of hMSC spheroids.

**Results:**

The acoustic levitation in anti-gravity bioreactor made spheroids faster and more compact compared to the conventional hanging drop method, which resulted in the upregulation of angiogenic paracrine factors of hMSCs, such as vascular endothelial growth factor and angiopoietin 2. Injected hMSCs spheroids cultured in the anti-gravity bioreactor exhibited improved therapeutic efficacy, including the degree of limb salvage, capillary formation, and attenuation of fibrosis and inflammation, for mouse hindlimb ischemia model compared to spheroids formed by the conventional hanging drop method.

**Conclusion:**

Our stem cell culture system using acoustic levitation will be proposed as a new platform for the future 3D cell culture system.

**Graphical Abstract:**

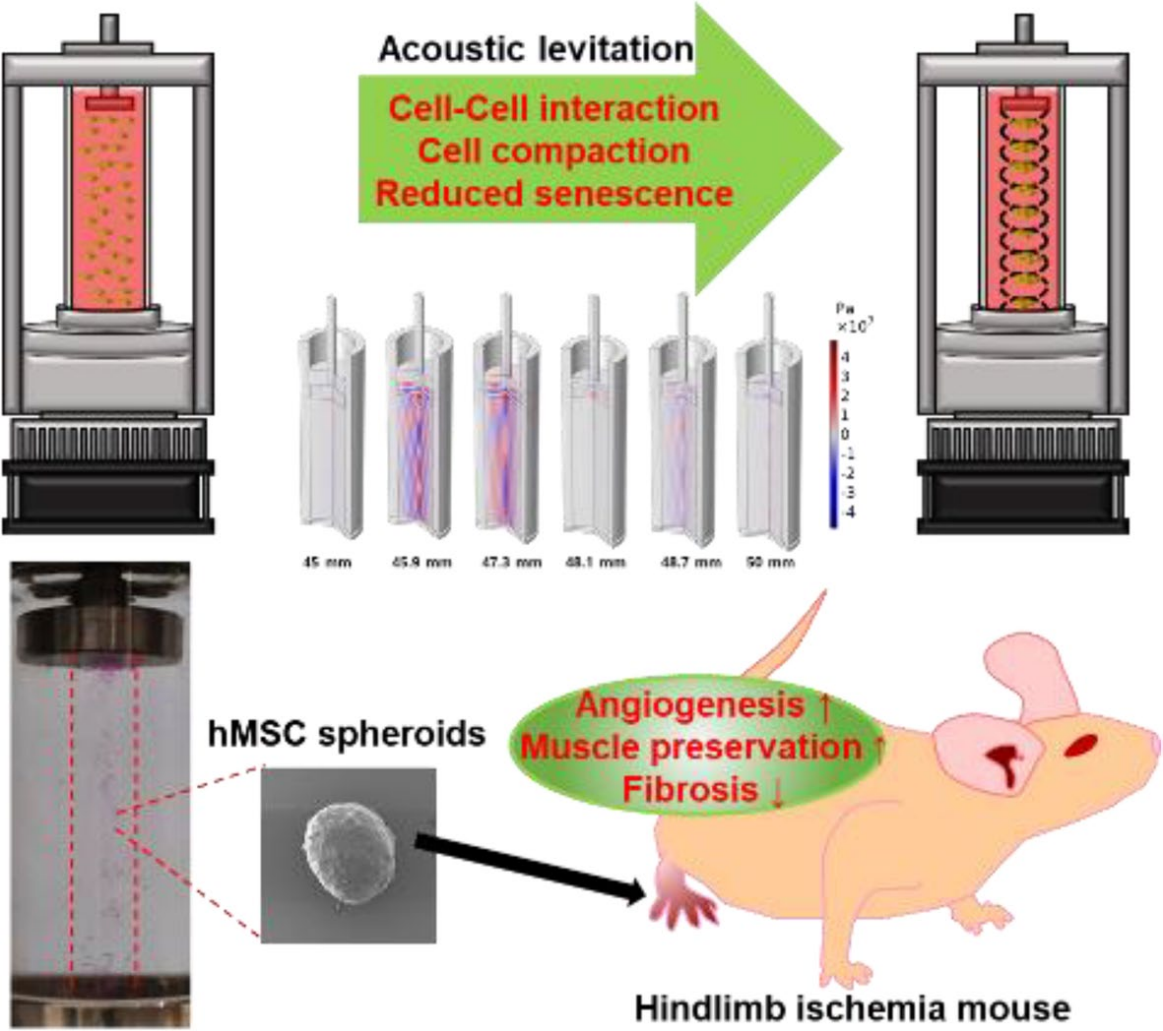

**Supplementary Information:**

The online version contains supplementary material available at 10.1186/s40824-023-00383-w.

## Introduction

Three-dimensional stem cell spheroids have gained a growing attention as cell therapies and biomaterials. So far, spheroids have been fabricated by numerous methods including pellet culture, hanging drop, spinner flask, magnetic levitation, and culture in space [1–4]. The conventional 3D culture methods can change the phenotypes of cultured cells such as gene expressions of proliferation, cell–cell interaction, and paracrine factors compared to 2D stem cells [1, 2]. The time for spheroid formation is basically long in most methods. From 2 to 7 days, there is a large deviation depending on the culture method and process. Even in the culture process, complex or labor-intensive processes are included. Pellet culture and hanging drop methods require separate preparation for each spheroid [1, 5], and spinner flask method require continuous rotational control [6, 7]. Methods using magnetic levitation require additional steps such as mechanical operation or nanoparticles that enter the medium for help levitation [8, 9]. To solve problems such as time and labor, the acoustic levitation was applied in this study.

**Fig. 1:**
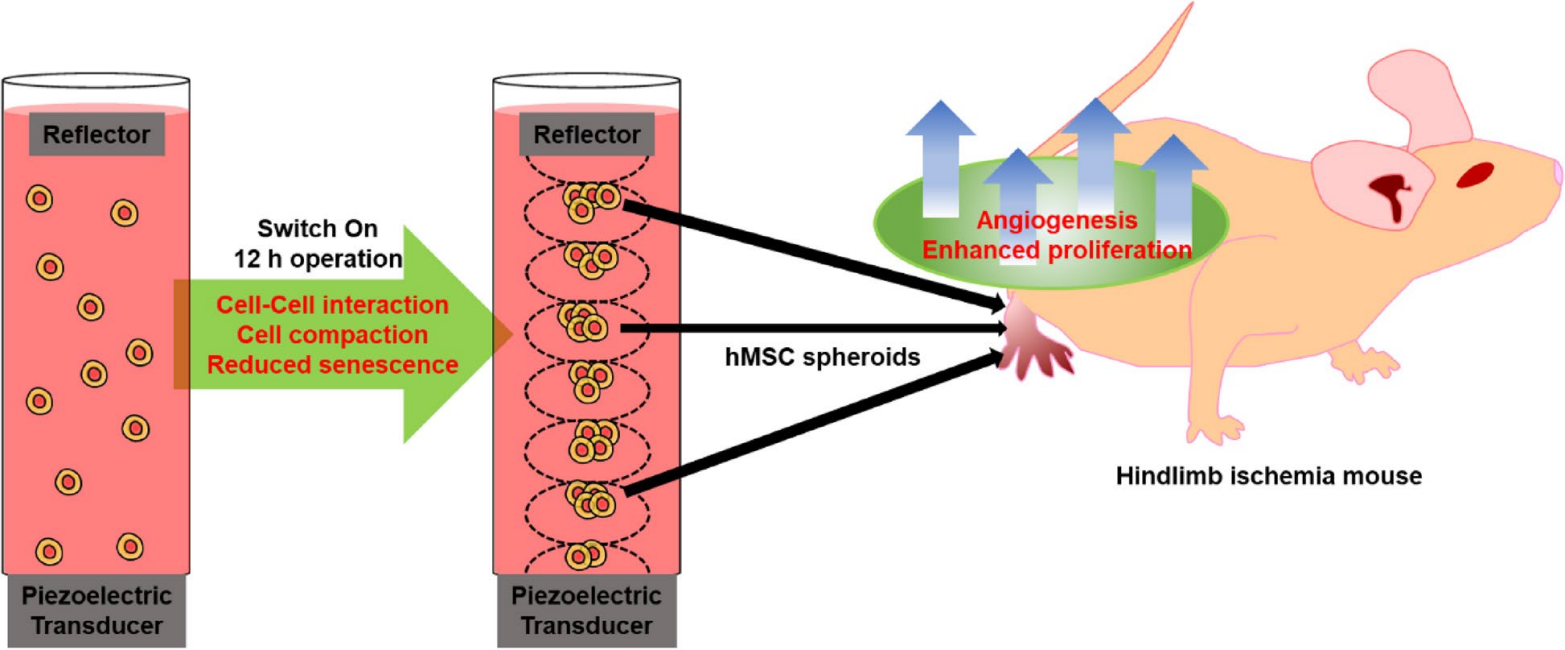
Schematic diagram of 3D stem cell spheroid culture by acoustic levitation. During incubation with acoustic levitation, hMSCs were aggregated near the node and incubated as spheroids. These spheroids showed the characteristics of spheroids such as improved expression of angiogenic factors and proliferation factors. By injecting these spheroids, the treatment efficiency of hind limb ischemia was observed

To solve these limitations, we cultured human mesenchymal stem cells (hMSCs) with the acoustic levitation method to fabricate spheroids. Several studies have developed an anti-gravity bioreactor equipped with transducer and reflector for acoustic levitation [10, 11]. The anti-gravity bioreactor only needs a simple process, one injection of the medium and cells before the operation. After that, the anti-gravity bioreactor does not require any additional control. Then, standing wave is generated into the liquid medium through the vibrator. The energy of the standing wave is transferred into the medium, creating a pressure field. The hMSCs in the medium were located in the node of the standing wave. The hMSCs are arranged and cultured while floating in the anti-gravity bioreactor. Spheroids are formed when cultured for more than 12 h in the anti-gravity bioreactor. (Fig. 1).

The anti-gravity bioreactor takes shorter time to formulate spheroids with compact and uniform morphologies compared to those of conventional methods without cytotoxicity. As previously reported, compact spheroids exhibited higher metabolic activity, viability, and proliferation compared to spheroids with voids and uneven morphologies [12]. As a result, after 12 h of incubation, the gene expression levels of angiogenic, senescent and proliferative factors were improved in the spheroids from the anti-gravity bioreactor compared to the conventional hanging drop method. Also, in in vivo experiment, spheroids formed in anti-gravity bioreactor showed remarkably improved therapeutic efficacy for the hindlimb ischemia mouse model compared to conventional spheroids formed by hanging drop method. Overall, the generation of spheroids via our anti-gravity bioreactor with acoustic levitation may pave the way for a new stem cell culture platform that overcomes the drawbacks of the conventional methods.

## Materials and methods

### Anti-gravity bioreactor fabrication

As the sound source of the anti-gravity bioreactor, NB-59S-09S having a resonance frequency of 1.6 MHz from TDK was used as the piezoelectric transducer. The transducer driving voltage was fixed at 12 V DC, and a 12 V-1.5A DC adapter was used as the power supply. For cooling the bioreactor, ShenzenAV's TEC1-12,708 (40 $\times$ 40 mm) 12 V-8A DC input was used as a Peltier device. All frames of the bioreactor were made of aluminum to maximize cooling efficiency. An aluminum disk (diameter 13 cm, thickness 5 mm) was applied for the acoustic reflector, and a rod with an M5 thread was attached to the rear to adjust the height of the reflector. A Pyrex glass tube (inner diameter 14 mm, thickness 3 mm, height 8 cm) was used as the outer wall of the bioreactor. An O-ring was used at the bottom to prevent leakage of the culture medium, and another O-ring was set at the upper lid to prevent contamination from outside and a hole (3 mm diameter) was punched on it to maintain aeration of the bioreactor.

### Cell culture

Human MSCs were purchased from Lonza (Allendale, NJ, USA). We cultured in Dulbecco's modified Eagle's medium (Gibco BRL, Grand Island, NY, USA) supplemented with 10% (v/v) fetal bovine serum (Gibco BRL) and 1% (v/v) penicillin/streptomycin (Gibco BRL). hMSCs at 5 to 6 passages were used for the experiments. Two-dimensional culture group were cultured on tissue culture plates. For the fabrication of spheroids by hanging drop method, 2.5 × 10^4^ cells in 35 μl of media were cultured for 12 or 24 h [5, 13]. For the fabrication of spheroids by anti-gravity bioreactor, 1 × 10^6^ cells were cultured in the device and incubated for 12 or 24 h. In the anti-gravity bioreactor, 50 ± 10 spheroids were formed. Photothermal images of the anti-gravity bioreactor were obtained using an infrared thermal imaging system for 24 h (FLIR E4, FLIR Systems Inc., USA).

### Spheroid characterization

Optical images of the spheroids were obtained using a bright field microscopy (CKX53, Olympus, Tokyo, Japan). The volume of the spheroids was calculated with the following equation: [volume = width × height^2^ × 0.5] [14].

### Spheroid shrinking and compaction analysis

The percentages of spheroid shrink were calculated with the following equation: [volume of additional culture for 12 h after spheroid formation / volume of additional culture for 0 h after spheroid formation]. The spheroid samples were fixed in a 1% glutaraldehyde solution for 1 h and washed three times with phosphate buffered saline (PBS, Gibco BRL) and dehydrated using graded ethanol solution from 50 to 100% (v/v). Then samples were dried for 5 min and visualized using scanning electron microscope (SEM, JSM-7500F, 30 kV, JEOL, Tokyo, Japan). To obtain slide sections of spheroids, the samples were embedded in OCT compound (SciGen Scientific, Gardenas, CA, USA) and cut into 10 μm-thickness. The sections were stained with RITC-conjugated phalloidin (Sigma-Aldrich, St. Louis, MO, USA) and 4′,6-diamidino-2-phenylindole (DAPI, Vector Laboratories, Burlingame, CA, USA). The fluorescent images of F-actin were visualized using a fluorescent microscope (DMi8, Leika, Wetzlar, Germany).

### Measurement of cell efficiency in anti-gravity bioreactor

The cell efficiencies of the spheroids were calculated with the following equation: [(1 × 10^6^ cells – the number of cells remaining in the medium after 12 h incubation) / 1 × 10^6^ cells] × 100.

### Measurement of cell viability

To visualize live and dead cells, FDA/EB (fluorescein/ethidium bromide, Sigma) solution was used. To evaluate cell viability, we used ApopTag® assay (Terminal deoxynucleotidyl transferase dUTP nick end labeling, TUNEL, Millipore, Bedford, MA, USA) was used. The cells stained with FDA/EB and assay were photographed by a fluorescence microscope (Leika).

### Gene expression analysis

To evaluate relative mRNA expression levels of human connexin 43 (Cx43), CASPASE-3, vascular endothelial growth factor (VEGF), insulin-like growth factor 1 (IGF-1), angiopoietin 2 (ANGPT2), p16, p21, proliferating cell nuclear antigen (PCNA), and mouse CD31 and alpha-smooth muscle actin (α-SMA), quantitative real-time polymerase chain reaction (qRT-PCR) was used 12 h after the culture in the anti-gravity bioreactor. Glyceraldehyde-3-phosphate dehydrogenase (GAPDH) was used as a housekeeping gene for normalization. The primer sequences are described in Supplementary Table 1. Total RNA was extracted from the samples using TRIzol reagent (Life Technologies, Inc., Carlsbad, CA, USA). Then the solution was mixed with chloroform and centrifuged at 10,000 g for 10 min. The supernatant was mixed with 80% (v/v) isopropanol and centrifuged 10,000 g for 10 min. RNA was then washed using 75% (v/v) ethanol. After discarding supernatant and subsequent drying, the samples were dissolved in DEPC water. For cDNA synthesis, AccuPower RT Premix (Bioneer, Daejeon, Korea) was used. After synthesis of cDNA from RNA samples, qRT-PCR was performed using the SsoAdvanced™ Universal SYBR green kit (Bio-Rad, Hercules, CA, USA).

### Western blot

The cell and tissue samples were lysed in cell lysis buffer (Abcam). Spheroids and ischemic limb muscles were collected and lysed and then centrifuged at 10,000 g for 10 min to separate debris. Proteins from cell and tissue samples were separated by SDS-PAGE and then transferred to a PVDF membrane (Bio-Rad) and labeled with primary antibodies against GAPDH, BCL-2, CD31, and α-SMA (these antibodies were purchased from Abcam, Cambridge, UK), and CASPASE-3 (Cell signaling Technology Inc., Danvers, MA, USA) overnight. Then the membrane samples were incubated with HRP-conjugated secondary antibody (R&D Systems, Minneapolis MN, USA) for 1 h at room temperature. The blots were visualized using X-ray film blue (Agfa HealthCare NV, Mortsel, Belgium), followed by the quantification using ImageJ software.

### Immunohistochemistry

For antigen retrieval, the spheroid and tissue sections were reacted with citrate solution for 20 min (10 mM, pH 6.0). Following nonspecific region blockade with normal goat serum (Sigma) and triton X, the sections were reacted with primary antibodies against E-cadherin, PCNA, CD31, and α-SMA (these antibodies were purchased from Abcam) overnight at 4 ℃. Then the samples were washed using PBS, followed by incubation with FITC-conjugated secondary antibody (Jackson Immuno Research Laboratories, West Grove, PA, USA) for 1 h at room temperature. For nucleus staining, DAPI was used for the samples. Lastly, the samples were photographed using a fluorescent microscope (Leika).

### Mouse hindlimb ischemia induction

Five-week-old female athymic mice (Orient, Seoul, Republic of Korea) were used for animal experiments. We anesthetized mice using Rompun (10 mg/kg) and ketamine hydrochloride (100 mg/kg) before surgery. All upstream femoral arteries were tied (Ethicon, Somerville, NJ, USA) and excised from their proximal origin as previously described [14]. To assess therapeutic efficacy, 1 × 10^6^ of cells or spheroids in 100 µL of PBS that consist of equivalent number of cells were injected after hindlimb ischemia modeling. All animal experiments were approved by the Institutional Animal Care and Use Committee of Sungkyunkwan University (SKKUIACUC 2019–03-24–2).

### Histology

Hematoxylin and eosin (H&E) staining (Sigma) was performed to confirm spheroids morphologies, muscle degeneration, and tissue inflammation. Masson’s trichrome and Picro Sirius Red staining (Sigma) were performed to assess tissue fibrosis and detect fibrillary collagen, respectively. The samples were photographed with a bright field microscope (Olympus).

### Statistical analysis

All data were presented as mean ± standard deviation. Statistical comparisons were conducted using Student’s *t* test or one-way analysis of variance (ANOVA) with Tukey’s significant difference post hoc test using SPSS software (SPSS Inc., USA). P value under was considered as significant difference.

## Results

### Theoretical calculations for designing the anti-gravity bioreactor

**Fig. 2:**
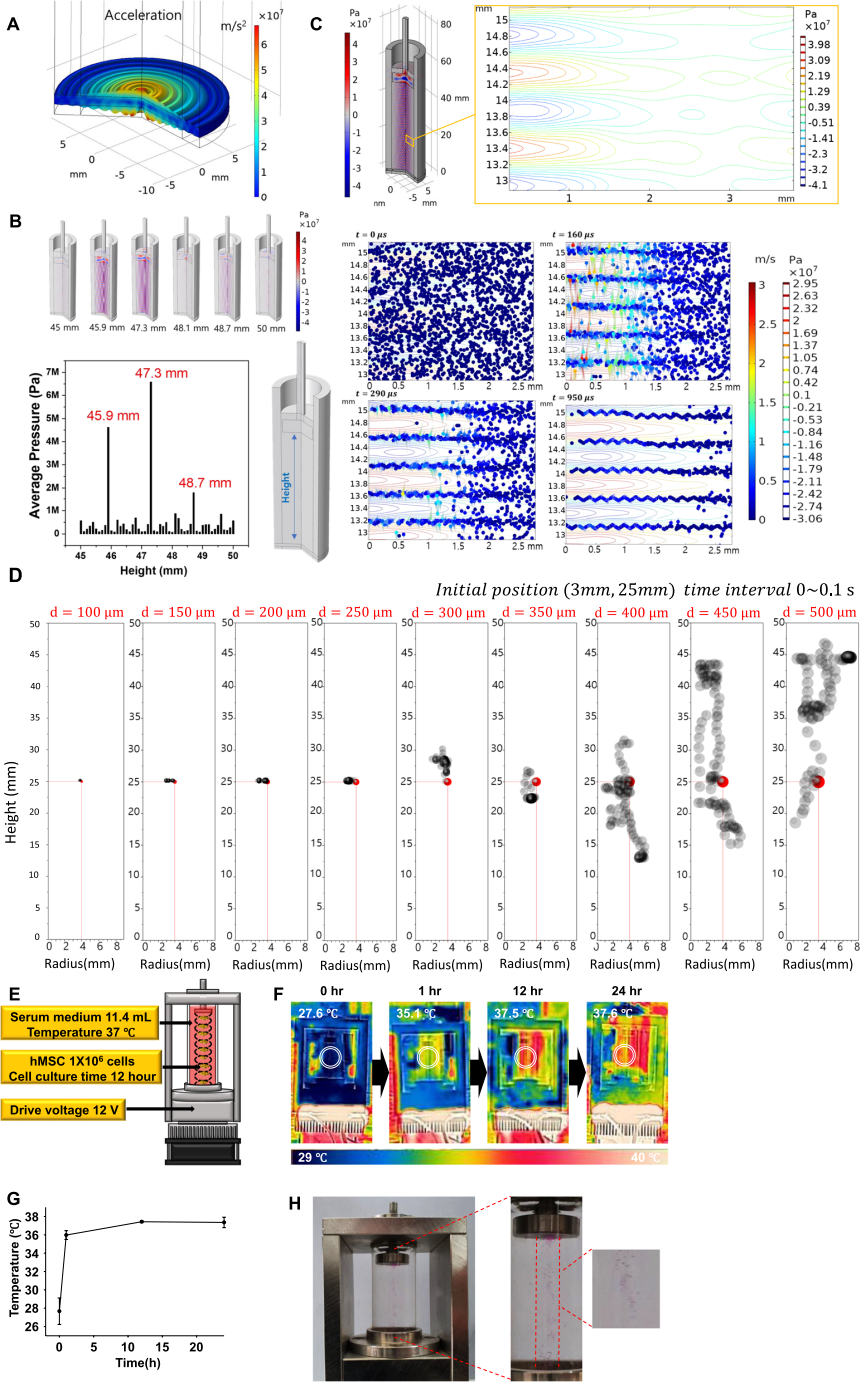
Characterization of anti-gravity bioreactor. **A** Calculated acceleration magnitude and relative deformation of a piezoelectric transducer used in this work **B** Acoustic pressure field as a function of height between reflector and actuator, the maximum average pressure field was obtained at 47.3 mm **C** Acoustic pressure contour plot of a selected zone (orange color rectangular solid box) and the trajectories of particles representing single cells over time there. **D** Trajectories of single particle for spheroid for 0.1 s under the given acoustic pressure field, the red point indicates the initial position of the particle. The range of the particle size is from 100 μm to 500 μm in diameter. **E** Cell culture conditions in anti-gravity device. **F** Temperature variation images and **G** quantitative data during 24 h of anti-gravity bioreactor operation adjusted with Peltier cooling device. H Representative images of hMSC spheroids cultured in the anti-gravity bioreactor

To design the anti-gravity bioreactor, theoretical calculations were conducted on the piezoelectric transducer, the geometry of the reactor, the standing wave resonance condition, the behavior of small particles in acoustic pressure field, and the limit of the particle size that can be stably levitated. A piezoelectric transducer that is an acoustic sound source of the anti-gravity bioreactor was used and it has the same specifications as in our previous paper [15]. Figure 2A shows the relative deformation and acceleration magnitude of the piezoelectric transducer activated at 12 V (peak to peak) with a resonant frequency 1.6 MHz. In the theoretical calculations, the piezoelectric transducer was placed at the bottom of the reactor of a circular tube shape (inner diameter 14 mm, thickness 3 mm) made of glass filled with water, and an aluminum acoustic reflector (diameter 13 mm, thickness 5 mm) was placed on the top. In addition, trajectories of many small particles (30 µm in diameter, 100,000 particles) representing single cells were calculated in order to observe the behavior of single cells after the formation of standing waves in anti-gravity bioreactor. And another trajectory calculations for each large particle size (100 ~ 500 µm) representing a spheroid were performed to determine the stability of levitation in the acoustic field. Detailed calculation conditions are shown in Supplementary Fig. 1.

The height *h* from the piezoelectric transducer to the reflector is a major variable in the formation of an acoustic standing wave in the anti-gravity bioreactor. Considering the resonance frequency of the piezoelectric actuator 1.6 MHz and the speed of sound waves in the culture medium of 1500 m/s, the period of the standing acoustic wave is approximately 468.75 µm. However, since the bioreactor do not have a perfectly symmetrical configuration, calculation of the acoustic standing wave formation according to *h* is required under the given geometrical structure of the bioreactor. Figure 2B shows the pressure field of an acoustic standing wave formed in the bioreactor when *h* is varied from 45 to 50 mm. Uniform and most high acoustic pressure fields throughout the bioreactor was obtained at 47.3 mm. The pressure distribution of the acoustic standing wave formed inside the bioreactor at the height condition of 47.3 mm is shown in Fig. 2C.

To predict the behavior of individual cells in the anti-gravity bioreactor, the trajectories of 30 μm diameter particles under the acoustic standing wave were calculated and the results are shown in Supplementary video 1 and at the bottom of Fig. 2C. Within 1 ms, all particles were trapped near nodes of the acoustic standing wave. Instantaneous state of motion of particles at 160 μs indicates that the pressure difference in the acoustic standing wave is the driving force for particle acceleration. The color of a particle represents the speed the particle has at any given moment. Also, the direction and length of a particle’s tail means magnitude and direction of acceleration, respectively.

When the cells trapped near the nodes of the acoustic standing wave in the anti-gravity bioreactor grow into spheroids with a size of several hundred micrometers over time, it is necessary to predict the size of the spheroid that can be stably levitated in a given acoustic standing wave. Particles with a diameter of 100 to 500 μm were placed at a certain position (3 mm radius, 25 mm height) in the anti-gravity bioreactor where the acoustic standing wave was formed, and the particle traces for 0.1 s were calculated and the results are shown in Fig. 2D. The trace distribution of particles under 250 μm did not deviate significantly from the initial position. However, it can be seen that particles larger than 300 μm move very violently throughout the entire bioreactor unstably out of the initial position. The calculation results of the temporal motion of 250 and 500 μm particles representing these two cases are shown in Supplementary video 2.

### Optimization of anti-gravity bioreactor operation

The anti-gravity bioreactor was driven at 12 V. 11.4 ml of medium and 1 × 10^6^ hMSCs were used for 12 h (Fig. 2E). During incubation, the temperature of the anti-gravity bioreactor was raised to around 36 ℃ in 1 h and maintained at around 37 ℃ until 24 h. The temperature range optimized for cell culture was not exceeded when the anti-gravity bioreactor was driven in the incubator. The temperature of the anti-gravity bioreactor was suitable for cell culture up to 24 h in cell culture (Fig. 2E, F). Most of the hMSCs were centralized within seconds when the anti-gravity bioreactor was turned on. The hMSCs floated and clumped together with adjacent hMSCs until the anti-gravity bioreactor stopped working. (Fig. 2H). Supplementary Fig. 2 showed the process of spheroids formation in anti-gravity bioreactor in a time-dependent manner.

### Characterization of spheroids

**Fig. 3:**
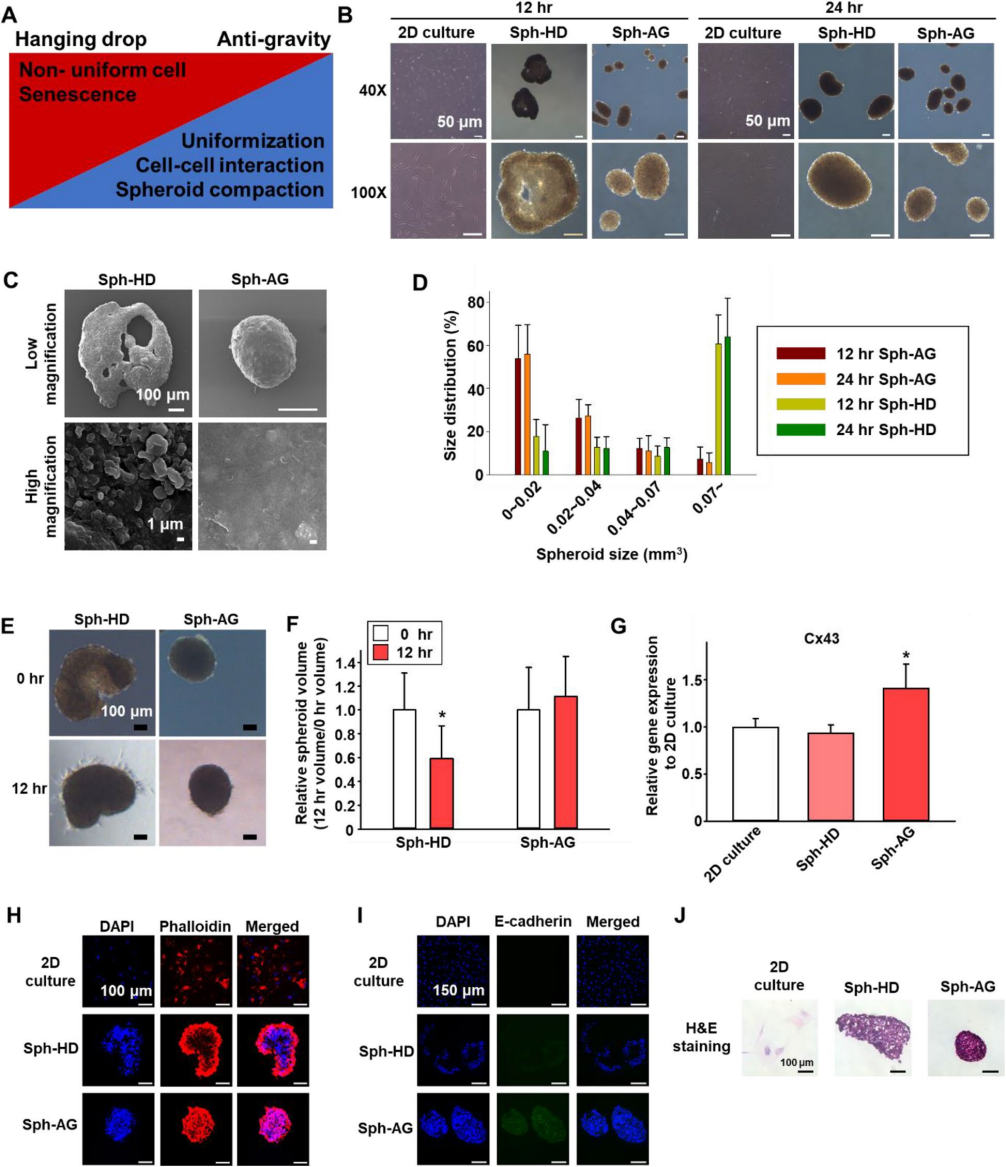
Characterization of hMSC spheroids cultured in anti-gravity bioreactor. **A** Characteristics of spheroids according to culture methods. **B** Representative images of hMSC cells and spheroids cultured by hanging drop or anti-gravity for 12 and 24 h. **C** Representative SEM images of hMSC spheroids at 12 h. **D** Size distribution of hMSC spheroids (*n* = 6). **E** Images of shrinking test. Scale bars = 100 μm. **F** Shrinking test was performed to confirm the spheroid compaction 12 h after the additional incubation on culture dish (*n* = 6, * *p* < 0.001 versus hanging drop group). **G** Relative Cx43 mRNA expression level of hMSCs evaluated by qRT-PCR (*n* = 6, * *p* < 0.05 versus all other group). **H** Fluorescent images of F-actin (red) in hMSCs. Scale bars = 100 μm. **I** e-cadherin by immunostaining were observed. **J** H&E staining images of hMSCs

The spheroids generated by the hanging drop (Sph-HD) and the anti-gravity bioreactor (Sph-AG) exhibited different characteristics (Fig. 3A). Sph-AG were denser than Sph-HD 12 and 24 h after the formation confirmed by optical microscope (Fig. 3B). Empty spaces were observed inside the Sph-HD method at 12 h, but not inside the Sph-AG. At 24 h, both spheroids were completely formed indicating that the spheroid formation time were faster in anti-gravity bioreactor than in hanging drop. SEM images showed that there were the empty spaces in Sph-HD at 12 h, but not in Sph-AG (Fig. 3C). Then we measured the size distribution of Sph-HD and Sph-AG at 12 and 24 h (Fig. 3D). When we cultured the same number of cells, 171.17 and 166 spheroids were formed by anti-gravity, respectively whereas 49 and 42.3 spheroids were formed by hanging drop, respectively. The average size of Sph-HD was larger than Sph-AG at 12 and 24 h.

To investigate whether the spheroids maintain their state, we further cultured the spheroids on a culture dish. The volume of Sph-HD was reduced by 59% of the original volume, however, the volume of Sph-AG was not significantly reduced 12 h after the additional incubation (Fig. 3E and F). Previous studies have shown that Cx43 [16, 17], F-actin and E-cadherin help gap junction between cells [18, 19]. Cx43 mRNA expression level of Sph-AG was higher than that of 2D culture and Sph-HD (Fig. 3G). The fluorescent and optical images of F-actin, E-cadherin, and H&E staining showed that the compactness and homogeneity of Sph-AG were higher than those of 2D culture and Sph-HD (Figs. 3H-J). Our data demonstrated that compact and stable spheroids can be produced in a short time by anti-gravity method rather than the conventional hanging drop method.

### Formation efficiency and viability of spheroids

**Fig. 4:**
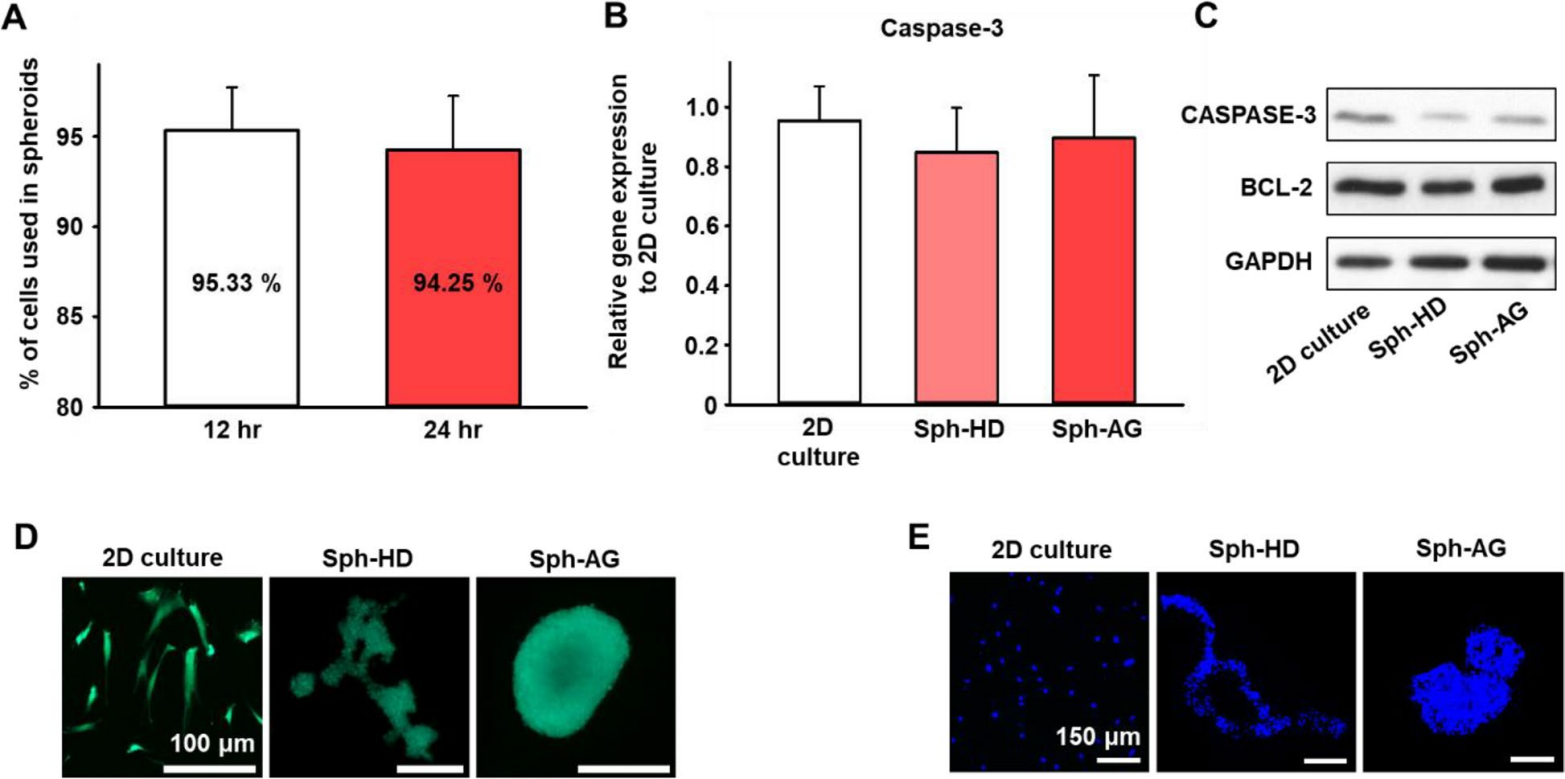
Spheroid formation efficiency and viability of hMSC spheroids cultured in anti-gravity bioreactor. **A** Spheroids formation efficiency in anti-gravity bioreactor at 12 and 24 h (*n* = 6). **B** Relative CASPASE-3 mRNA expression levels of hMSCs evaluated by qRT-PCR (*n* = 6). **C** Western blot analysis of hMSCs for apoptotic (CASPASE-3) and anti-apoptotic (BCL-2) factors in hMSCs at 12 h. **D** Fluorescent images of live (green) and dead (red) hMSCs using FDA/EB assay at 12 h. Scale bars = 100 μm. **E** Representative images of TUNEL (red)-positive apoptotic hMSCs at 12 h. Scale bars = 150 μm

To investigate the spheroid formation efficiency, we incubated 1 $\times$ 10^6^ hMSCs in the anti-gravity bioreactor for 12 and 24 h. In anti-gravity bioreactor, 95.33% and 94.25% of the hMSCs were formed as spheroids at 12 and 24 h, respectively (Fig. 4A). CASPASE-3 is a gene involved in cell apoptosis process [20, 21]. There was no significant difference in the gene expression level of CASPASE-3 in all groups at 12 h (Fig. 4B). The protein expression levels of CASPASE-3 and BCL-2 did not change in the spheroids compared to 2D culture (Fig. 4C). FDA/EB and TUNEL assay were used to visualize the viability of spheroids at 12 h (Figs. 4D and E). The fluorescent images showed that there were no dead cells both in Sph-HD and Sph-AG. Overall, our anti-gravity bioreactor could fabricate spheroids quickly without a notable toxicity.

### Phenotype changes of spheroids in anti-gravity bioreactor

**Fig. 5:**
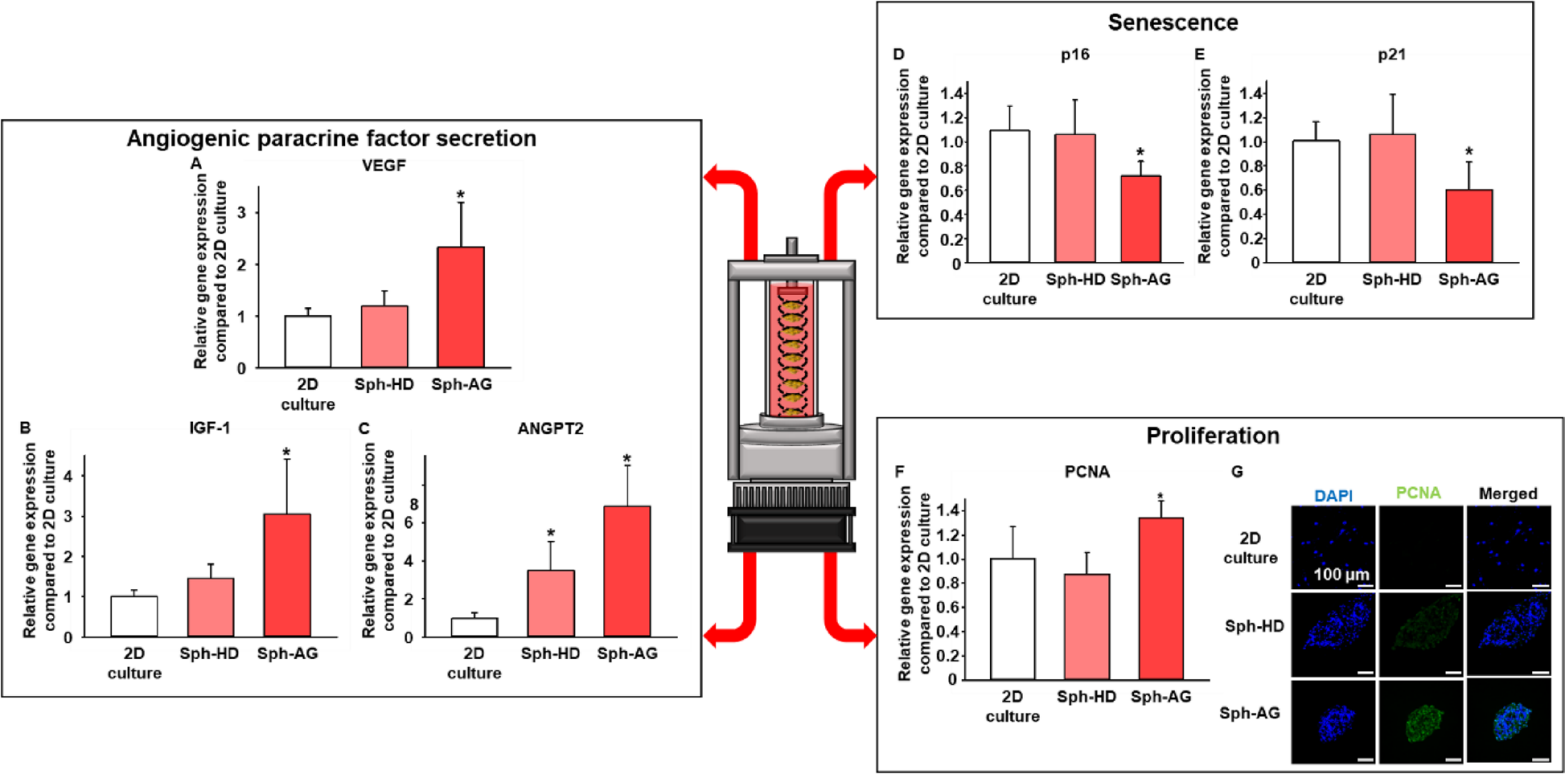
Phenotype changes of hMSC sphroids cultured in anti-gravity bioreactor at 12 h. Relative angiogenic mRNA expression levels of **A** VEGF, **B** IGF-1, and **C** ANGPT2 in hMSCs. Relative senescent mRNA expression levels of **D** p16 and **E** p21 in hMSCs. Relative proliferative mRNA expression level of **F** PCNA in hMSC. mRNA expression was evaluated by qRT-PCR (*n* = 6, * *p* < 0.05 versus all other group). **G** Representative images of immunostaining for PCNA (green) in hMSCs. Scale bars = 100 μm

To determine whether the spheroid formation by anti-gravity bioreactor changes the phenotype of hMSCs, we measured mRNA expression levels of angiogenic paracrine factors, senescence- and proliferation-related factors by qRT-PCR at 12 h. The mRNA expression levels of the angiogenic paracrine factors, including VEGF, IGF-1, and ANGPT2, which contribute to angiogenesis, cell survival [22, 23], proliferation, host cell migration [22, 24], and homeostasis [25, 26] were increased in the Sph-AG group compared to the other group (Figs. 5A-C). The mRNA expression levels of senescence-related factors, which were identified as p16 and p21 [27, 28], were significantly decreased in the Sph-AG group compared to the other group (Figs. 5D and E). The mRNA expression level of PCNA, which is associated with cell proliferation [29, 30], were significantly increased in Sph-AG group compared to the other group (Fig. 5F). Fluorescent images also showed that the protein expression of PCNA was higher in the Sph-AG group than the other group (Fig. 5G). All factors expressed in the Sph-HD group were not different from 2D culture. Taken together, the rapid formation of spheroids in our anti-gravity bioreactor elicited the upregulation of therapeutic paracrine factors compared to the conventional hanging drop method.

### Enhanced therapeutic effect of hind limb ischemia by anti-gravity bioreactor

**Fig. 6:**
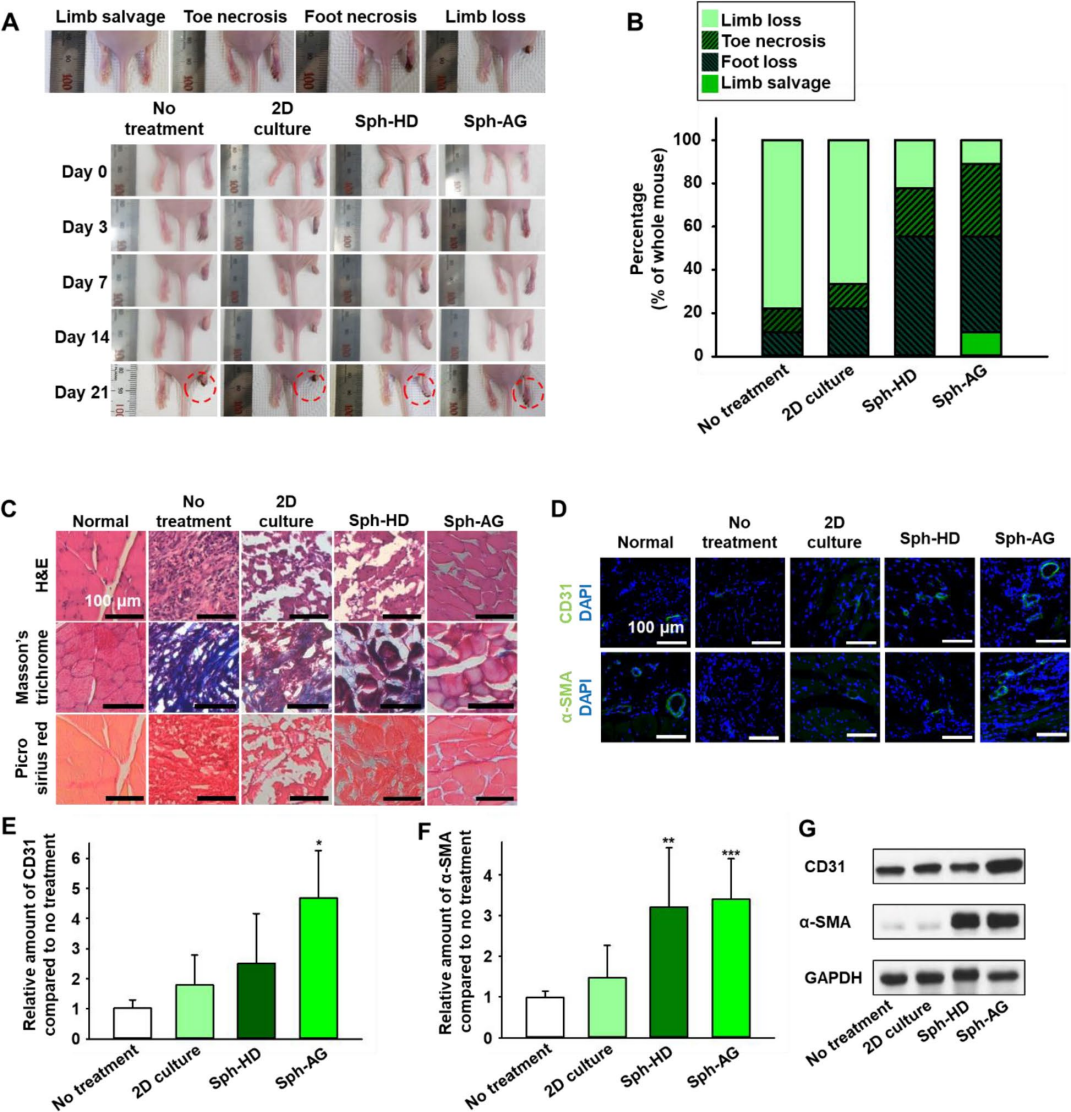
Therapeutic efficacy of hMSC spheroids in hindlimb ischemia mouse model. **A** Representative images of hindlimb ischemia mouse model 0, 3, 7, 14, and 21 days after treatment. **B** Limb salvage ratio 21 days after treatment (*n* = 10 per group). **C** Representative images of H&E, Masson’s trichrome, and Picro Sirius 21 days after treatment. Scale bars = 100 μm. **D** Representative immunohistochemical images of CD31 and SM-α (green) showing micro-blood vessels (day 21). Scale bars indicate 100 μm. The relative amount of **E** CD31 and **F** α-SMA were measured by qRT-PCR (*n* = 6, *p < 0.05 versus all other group, **p < 0.05 versus all other group except anti-gravity group, ***p < 0.05 versus all other group except hanging drop group) and **G** western blot analysis

To verify the therapeutic efficacy of spheroids, we injected Sph-HD and Sph-AG into the ischemic hindlimb 12 h after the culture. The representative images of hindlimb ischemia model treatment are shown in Fig. 6A. At day 21 after the surgery, we quantified the degree of limb salvage (Fig. 6B). The no treatment group showed no limb salvage at day 21, however, foot loss, toe necrosis, and limb loss were 11.1%, 11.1%, and 77.8% in the no treatment group, respectively. There were 22.2% of foot loss, 11.1% of toe necrosis, and 66.7% of limb loss in the 2D culture group. The Sph-HD treated group showed 55.6% of foot loss, 22.2% of toe necrosis, and 22.2% of limb loss. The Sph-AG treated group showed 11.1% of limb salvage, 44.4% of foot loss, 33.3% of toe necrosis, and 11.1% of limb loss. H&E, Masson’s trichrome, Picro sirius red staining images showed that the cell and muscle structure were preserved, and less inflammation and fibrosis occurred in the Sph-AG group compared to the other treatment group (Fig. 6C). To verify whether increased angiogenic factors in Sph-AG contribute to angiogenesis in hindlimb ischemia, we stained CD31 and and α-SMA (Fig. 6D). The gene and protein expressions of CD31 and αSMA in the Sph-AG group were significantly higher than the no treatment group (Figs. 6E-G).

## Discussion

Many methods for culturing 3D spheroids have been studied for decades. Conventional methods are represented by a method using magnetic field, hanging drop, aggrewell, and rotating cells [1–4]. The spheroids formed by these methods have been known to improve intercellular interactions, proliferative capacity, and gene expression [1, 2]. However, the conventional methods have two limitations. One is that the formation time of spheroids in the culture process takes at least 2 days [5–7, 9, 12, 31]. In addition, a complicated device or a labor-intensive process is required for the culture process. In a case of hanging drop method, setting for incubation is necessary drop by drop [1, 5]. Methods that utilize electromagnetic fields or use rotation of cells require the arrangement of complex devices for culturing cells. And it is necessary to fine-tune the rotational speed, arrangement of cells [6–9]. To overcome these limitations, we used an anti-gravity bioreactor using acoustic levitation method.

The anti-gravity bioreactor was designed based on the levitation by a one-dimensional standing wave from a single acoustic source. Therefore, the bioreactor has a cylindrical shape with a piezoelectric transducer as a sound source at the bottom and a reflector at the top. Since the natural frequency of the transducer and the density of the cell medium are fixed constants, the only variable for the formation of an acoustic standing wave is the spacing between the reflector and the actuator. In theory, a standing wave occurs if the spacing is an integer multiple of half the acoustic wavelength. In consideration of the volume of medium required for cell culture, the appropriate spacing for the acoustic standing wave was estimated within 45 to 50 mm. through computational calculation. As a result of the calculation, the most uniform and largest amplitude standing wave was formed at a spacing of 47.3 mm.

Given the optimal acoustic standing wave conditions, it was calculated how fast single cells were trapped; the trapping of cells into the node was completed within 1 ms. This is the first step in the formation of spheroids, and it can be understood as the effect of bringing the cells injected into the bioreactor close to each other near the nodes. Meanwhile, the cells trapped in the nodes grow into spheroids over time, however their size is limited by the dimensional size of a given acoustic standing wave. Theoretically, in order to predict the size of the spheroid allowed in the acoustic standing wave of the present work, the size of the particle in which the acoustic levitation can be stably maintained was calculated. Among the particles of 100 to 500 μm diameter set in the calculation, it was found that particles with 300 μm diameter or larger did not maintain acoustic levitation, and those with less than 300 μm were levitated as stable.

Based on these theoretical calculation results, the actual anti-gravity bioreactor was manufactured. Since temperature maintenance is important during cell culture, the material of the holder of the piezoelectric transducer, which is a heat source, is made of aluminum, and the temperature can be controlled by using a Peltier cooler at the bottom. In addition, the power source of the piezoelectric transducer’s driving circuit was supplied from the outside of the incubator to minimize the volume of the bioreactor.

11.4 ml medium was used to match the previously optimized reflector and transducer distance 47.3 mm. The number of hMSCs optimized by subsequent experiments was 1 × 10^6^ cells and the incubation time was 12 h. 12 V is supplied for the operation of the anti-gravity bioreactor (Fig. 2E). The transducer generates heat during operation [32, 33]. This heat raises the temperature of the medium. A fan was attached to the bottom of the transducer to avoid temperature increase during cell culture in this study. The optimal temperature without damage in the cell culture was around 37 °C [34]. Anti-gravity bioreactor increased to 36 °C within 1 h of operation. And most of the incubation time was maintained at around 37 °C (Figs. 2F and G). Therefore, there was no damage to cells due to temperature during use of the device. During operation, the acoustic levitation causes the cells to be cultured in a row in the center (Fig. 2H).hMSCs were cultured for 12 or 24 h, which is shorter than the conventional methods. Incompletely filled spheroids had a hollow part in the middle or were very large, so the inside was less aggregated. The size of the spheroids differed depending on the culture method rather than the incubation time. When cultured by hanging drop, the size was significantly larger than that of the spheroids cultured in the anti-gravity bioreactor (Fig. 3C). The size of the spheroids cultured with hanging drop was significantly larger because the inside was not filled like the previous optical images. In the low magnification SEM image, the middle of spheroid was not completely filled in the hanging drop group like the previous data. In the SEM image at high magnification, many hMSCs were not completely aggregated on the surface of the spheroid. In the spheroids cultured in the anti-gravity bioreactor, hMSCs were completely aggregated at both low and high magnifications (Fig. 3D). After harvesting, it was confirmed whether additional aggregation of hMSCs occurred in additional culture. In the Sph-HD, the hMSCs were compressed into the empty spaces inside and the size was reduced after 12 h. However, the size of Sph-AG group did not change after the spheroids were completely aggregated even before additional incubation (Figs. 3E and F). Sph-AG were formed within 12 h, but the Sph-HD were not complete spheroids until 12 h. Gap junction between cells and intercellular adhesion were evaluated with Cx43, F-actin, and E-cadherin. The expression of Cx43 was significantly elevated only in spheroids cultured in the anti-gravity bioreactor (Fig. 3G). Elevated Cx43 expression leads to reinforcement of gap junctions. The distribution of F-actin was confirmed by phalloidin staining, and the expression of E-cadherin was confirmed by immunostaining. The colors of phalloidin and E-cadherin appeared bright in the entire spheroid cultured in the anti-gravity bioreactor (Figs. 3H and I). The increase in F-actin distribution and E-cadherin expression is a phenomenon that appears as results of increased cell–cell adhesion. Therefore, the cell–cell adhesion was relatively well established in the anti-gravity bioreactor group compared to the other group. H&E staining images also confirmed that the arrangement of hMSCs was more compact in the anti-gravity bioreactor group (Fig. 3J). In anti-gravity group, all factors related to the density of spheroids were increased.

When cultured in the anti-gravity bioreactor for 12 h, 95.33% of the hMSCs were used as spheroids, and when cultured for 24 h, 94.25% or cells were used (Fig. 4A). There was no difference in the formation efficiency of the cells used as spheroids compared to the conventional spheroid studies. To investigate whether the acoustic levitation exhibited the cytotoxicity, we confirmed the viability of hMSCs by several methods. The expression of CASPASE-3, a pro-apoptosis factor, was checked to determine the damage to hMSCs. There was no difference in the expression of CASPASE-3 among all groups and there was no toxicity induced by the acoustic levitation (Fig. 4B). The expression of CASPSAE-3 and BCL-2, a pro-apoptosis factor and anti-apoptosis factor was also confirmed by western blot [35, 36]. The CASPASE-3 and BCL-2 data showed by western blot also supported the absence of toxicity (Fig. 4C). EB^+^ cells were not detected, and there was no dead signal in TUNEL assay (Figs. 4D and E).

There were genes which expression was changed in the spheroids formed in the anti-gravity bioreactor faster than the conventional methods. VEGF, an angiogenic factor frequently secreted from spheroids, was secreted more in the anti-gravity bioreactor group than in other groups (Fig. 5A). In addition, other factors related to angiogenesis, IGF-1, and ANGPT2, were also significantly increased in the anti-gravity bioreactor group (Figs. 5B and C). Although the formation time was short, an increase in angiogenic factor, which is one of the characteristics of spheroids, was confirmed. It is also confirmed that the expression levels of p16 and p21 were decreased, and PCNA was increased in Sph-AG group, indicating that the cells in Sph-AG might secrete paracrine factors continuously with maintaining proliferation and avoiding senescence (Figs. 5D-G). Further study is needed to reveal the mechanisms of the upregulation of these factors, which may be attributed to the rapid formation of cell–cell interaction in the Sph-AG group.

The treatment effects of spheroids were confirmed by the injection into hindlimbs of mice after surgery. Therapeutic efficacy was classified into 4 categories with the naked eyes: limb salvage, toe necrosis, foot necrosis, and limb loss [37]. In the NT and 2D culture groups, there the most of mice underwent limb loss. In contrast, the Sph-HD and Sph-AG groups blocked the progression of limb loss (Figs. 6A and B). Furthermore, some of the mice in the Sph-AG group showed limb salvage after the injection. Inflammation and muscle degeneration were observed in all groups except the Sph-AG group. However, the muscles were regenerating like the normal group in the Sph-AG group (Fig. 6C). Muscle degeneration was confirmed in H&E staining. Inflammation was confirmed by purple in H&E staining, blue in Masson’s trichrome staining and red in picro sirius red staining. The gene and protein expression levels of CD31 and α-SMA, which are factors involved in vascular regeneration, were also increased in the Sph-AG group compared to no treatment group (Figs. 6D-G). Taken together, upregulated paracrine factors in Sph-AG group elicited enhanced therapeutic efficacy in mouse hindlimb ischemia model.

## Conclusion

The goals of 3D stem cell spheroid culture study were to shorten the culture time and change the culture method to a simple one. In this study, we applied the acoustic levitation method for 3D stem cell spheroid culture. In our simple culture method, anti-gravity bioreactor formed stable stem cell spheroids in 12 h with enhanced angiogenic factors and decreased senescence factors. Acoustic levitation solved the limitations of 3D cell culture and suggested a new direction for 3D cell culture platform.

## Supplementary Information

**Additional file 1:** **Supplementary Table 1.** Primer sequence for each gene.

**Additional file 2:  Supplementary Fig. 1.** A COMSOL Multiphysic 5.5 with acoustic and particle tracing modules was used for the calculation. All dimensions and materials applied to acoustic pressure and particle tracing calculations were identical to the experimental cell culture vessel. In particle tracing calculations for single cells and a spheroid, the released particle properties were described like above.

**Additional file 3:  Supplementary Fig. 2.** Spheroid formation in the anti-gravity bioreactor according to incubation time.

**Additional file 4:  Supplementary video 1.** The video clip for initial 1 ms about the trajectories of a lot of particles representing single cells under the given acoustic pressure field.

**Additional file 5:  Supplementary video 2.** The video clips for initial 0.1 s about the trajectories of two particlesrepresenting different sized spheroids under the given acoustic pressure field. The 250 μm particle slightly moves and then stays at a stable position, however the 500 μm particle never stay at any stable position for 0.1s.

## Data Availability

The datasets used and/or analyzed during the current study are available from the corresponding author on reasonable request.

## Abbreviations

*hMSC*: Human mesenchymal stem cell
*PBS*: Phosphate buffered saline
*SEM*: Scanning electron microscope
*DAPI*: 4′,6-Diamidino-2-phenylindole
*FDA/EB*: Fluorescein/ethidium bromide
*TUNEL*: Terminal deoxynucleotidyl transferase dUTP nick end labeling
*Cx43*: Connexin 43
*VEGF*: Vascular endothelial growth factor
*IGF-1*: Insulin-like growth factor 1
*ANGPT2*: Angiopoietin 2
*PCNA*: Proliferating cell nuclear antigen
*α-SMA*: Alpha-smooth muscle actin
*qRT-PCR*: Quantitative real time-polymerase chain reaction
*GAPDH*: Glyceraldehyde-3-phosphate dehydrogenase
*H&E*: Hematoxylin and eosin
*Sph-HD*: Spheroids generated by the hanging drop
*Sph-AG*: Spheroids generated by the anti-gravity bioreactor
