# Supplementary material for: Subaqueous 3D stem cell spheroid levitation culture using anti-gravity bioreactor based on sound wave superposition
Source: Biomater Res. 2023 May 19;27:51. doi: 10.1186/s40824-023-00383-w (PMC10197840; doi:10.1186/s40824-023-00383-w)
Supplement: Supplementary file 1 — Additional file 1: Supplementary Table 1. Primer sequence for each gene. [file 40824_2023_383_MOESM1_ESM.docx]

| Gene | Primer (Forward) | Primer (Reverse) |
| --- | --- | --- |
| Species | **Human** | |
| Cx43 | ACT GGC GAC AGA AAC AAT TCT TC | TTC TGC ACT GTA ATT AGC CCA GTT |
| CASPASE-3 | CCT GGT TAT TAT TCT TGG CGA AA | GCA CAA AGC GAC TGG ATG AA |
| VEGF | GAG GGC AGA ATC ATC ACG AAG T | CAC CAG GGT CTC GAT TGG AT |
| IGF-1 | CAC AGA CGG GCA TCG TGG AT | ACT TGG CAG GCT TGA GGG GT |
| ANGPT2 | AAG AGA TCA AGG CCT ACT GTG ACA | TCC TCA CGT CGC TGA ATA ATT G |
| p16 | GTG GAC CTG GCT GAG GAG | CTT TCA ATC GGG GAT GTC TG |
| PCNA | AGG GCT GAA GAT AAT GCT GAT ACC | CTC CTG TTC TGG GAT TCC AAG TTG |
| Species | **Mouse** | |
| CD31 | GTG GTG CTG ATG TCC ACA AG | AAC AGT GTC TGC CAT CCT TCT |
| a-SMA | GCT ACT TAC CCT GAC AGC GA | CTT GTT TGG GAA GCG AGT GG |

**Supplementary table 1.** Primer sequence for each gene.
