# Supplementary material for: Subaqueous 3D stem cell spheroid levitation culture using anti-gravity bioreactor based on sound wave superposition
Source: Biomater Res. 2023 May 19;27:51. doi: 10.1186/s40824-023-00383-w (PMC10197840; doi:10.1186/s40824-023-00383-w)
Supplement: Supplementary file 2 — Additional file 2: Supplementary Fig. 1. A COMSOL Multiphysic 5.5 with acoustic and particle tracing modules was used for the calculation. All dimensions and materials applied to acoustic pressure and particle tracing calculations were identical to the experimental cell culture vessel. In particle tracing calculations for single cells and a spheroid, the released particle properties were described like above. [file 40824_2023_383_MOESM2_ESM.docx]

Supporting Information

**Subaqueous 3D stem cell spheroid levitation culture using anti-gravity bioreactor based on sound wave superposition**

Jung Hwan Park^1,†^, Ju-Ro Lee^2,†^, Sungkwon Park^3,†^, Yu-Jin Kim^1^, Jeong-Kee Yoon^4^, Hyun Su Park^1^, Jiyu Hyun^1^, Yoon Ki Joung^2,5^, Tae Il Lee^6,*^, and Suk Ho Bhang^1,*^

1School of Chemical Engineering, Sungkyunkwan University, Suwon 16419, Republic of Korea

^2^Center for Biomaterials, Biomedical Research Institute, Korea Institute of Science and Technology, Seoul, 02792, Republic of Korea

^3^Department of Food Science and Biotechnology, College of Life Science, Sejong University, Seoul 05006, Korea.

^4^Department of Systems Biotechnology, Chung-Ang University, Anseong-si, Gyeonggi-do, 17540, Republic of Korea

^5^Division of Bio-Medical Science and Technology, University of Science and Technology, Republic of Korea, Seoul, 02792, Republic of Korea

^6^Department of Materials Science and Engineering, Gachon University, Seongnam-si, Gyeonggi-do, 13120, Republic of Korea

†These Authors contributed equally to this work.

*Correspondence: t2.lee77@gachon.ac.kr, sukhobhang@skku.edu


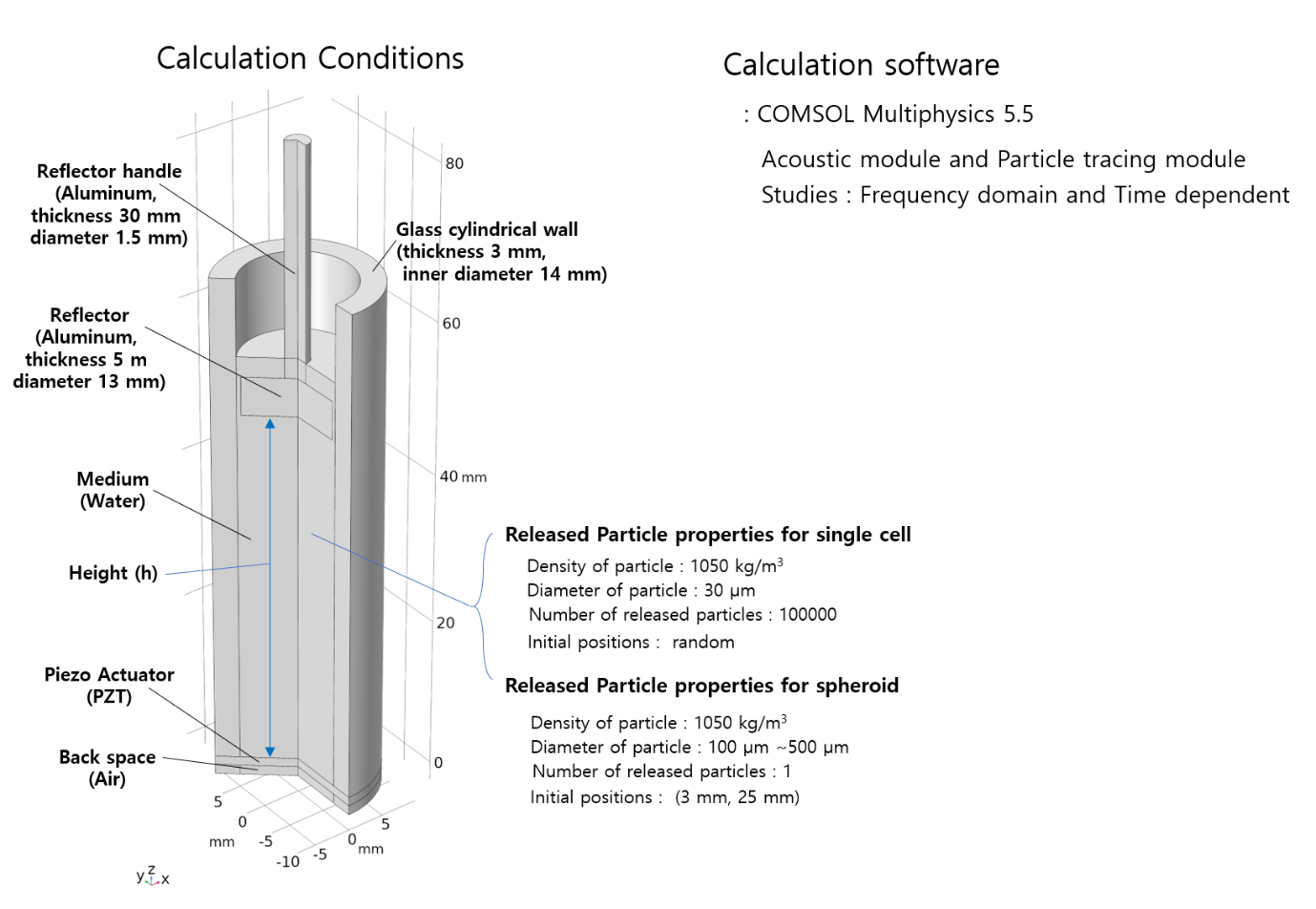


**Supplementary Fig. 1.** A COMSOL Multiphysic 5.5 with acoustic and particle tracing modules was used for the calculation. All dimensions and materials applied to acoustic pressure and particle tracing calculations were identical to the experimental cell culture vessel. In particle tracing calculations for single cells and a spheroid, the released particle properties were described like above.
