# Supplementary material for: Subaqueous 3D stem cell spheroid levitation culture using anti-gravity bioreactor based on sound wave superposition
Source: Biomater Res. 2023 May 19;27:51. doi: 10.1186/s40824-023-00383-w (PMC10197840; doi:10.1186/s40824-023-00383-w)
Supplement: Supplementary file 3 — Additional file 3: Supplementary Fig. 2. Spheroid formation in the anti-gravity bioreactor according to incubation time. [file 40824_2023_383_MOESM3_ESM.docx]

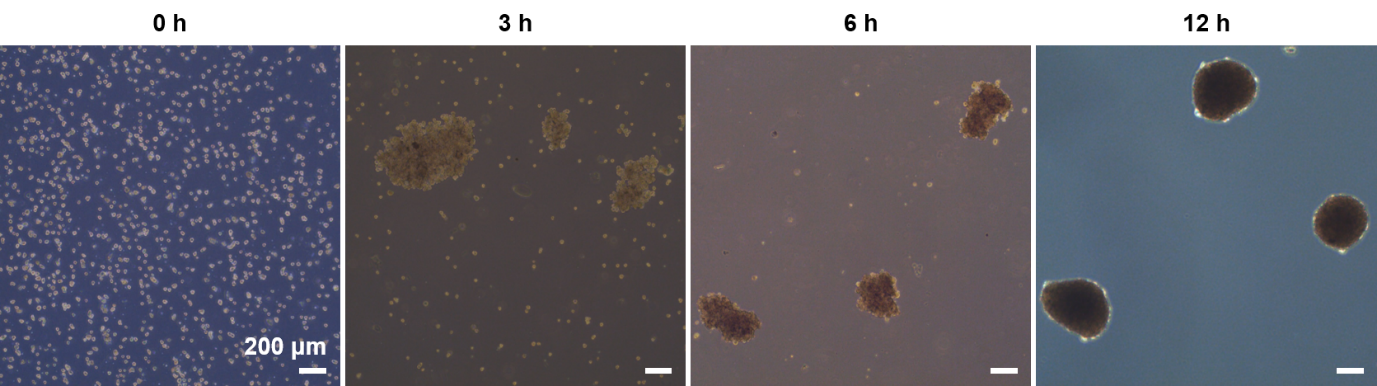


**Supplementary Fig. 2.** Spheroid formation in the anti-gravity bioreactor according to incubation time.
