# Supplementary material for: Subaqueous 3D stem cell spheroid levitation culture using anti-gravity bioreactor based on sound wave superposition
Source: Biomater Res. 2023 May 19;27:51. doi: 10.1186/s40824-023-00383-w (PMC10197840; doi:10.1186/s40824-023-00383-w)
Supplement: Supplementary file 4 — Additional file 4: Supplementary video 1. The video clip for initial 1 ms about the trajectories of a lot of particles representing single cells under the given acoustic pressure field. [file 40824_2023_383_MOESM4_ESM.docx]

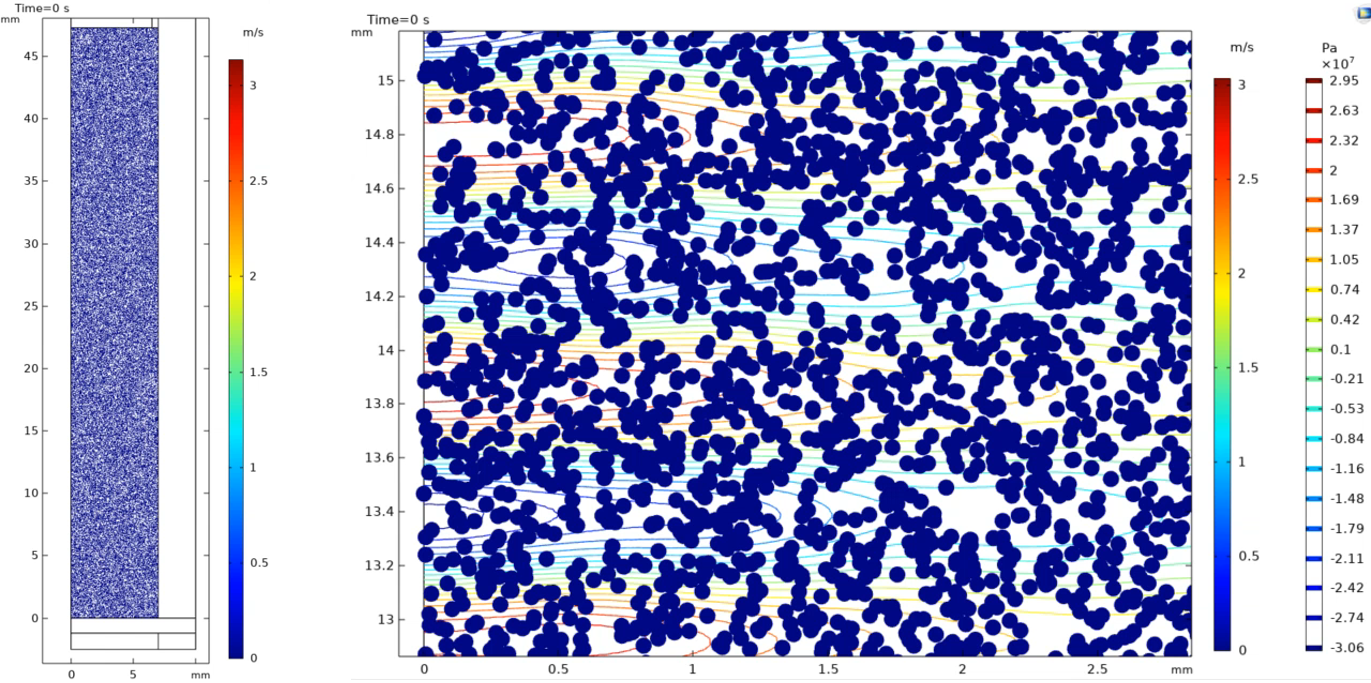


**Supplementary video 1.** The video clip for initial 1 ms about the trajectories of a lot of particles representing single cells under the given acoustic pressure field.
