# Supplementary material for: Subaqueous 3D stem cell spheroid levitation culture using anti-gravity bioreactor based on sound wave superposition
Source: Biomater Res. 2023 May 19;27:51. doi: 10.1186/s40824-023-00383-w (PMC10197840; doi:10.1186/s40824-023-00383-w)
Supplement: Supplementary file 5 — Additional file 5: Supplementary video 2. The video clips for initial 0.1 s about the trajectories of two particlesrepresenting different sized spheroids under the given acoustic pressure field. The 250 μm particle slightly moves and then stays at a stable position, however the 500 μm particle never stay at any stable position for 0.1s. [file 40824_2023_383_MOESM5_ESM.docx]

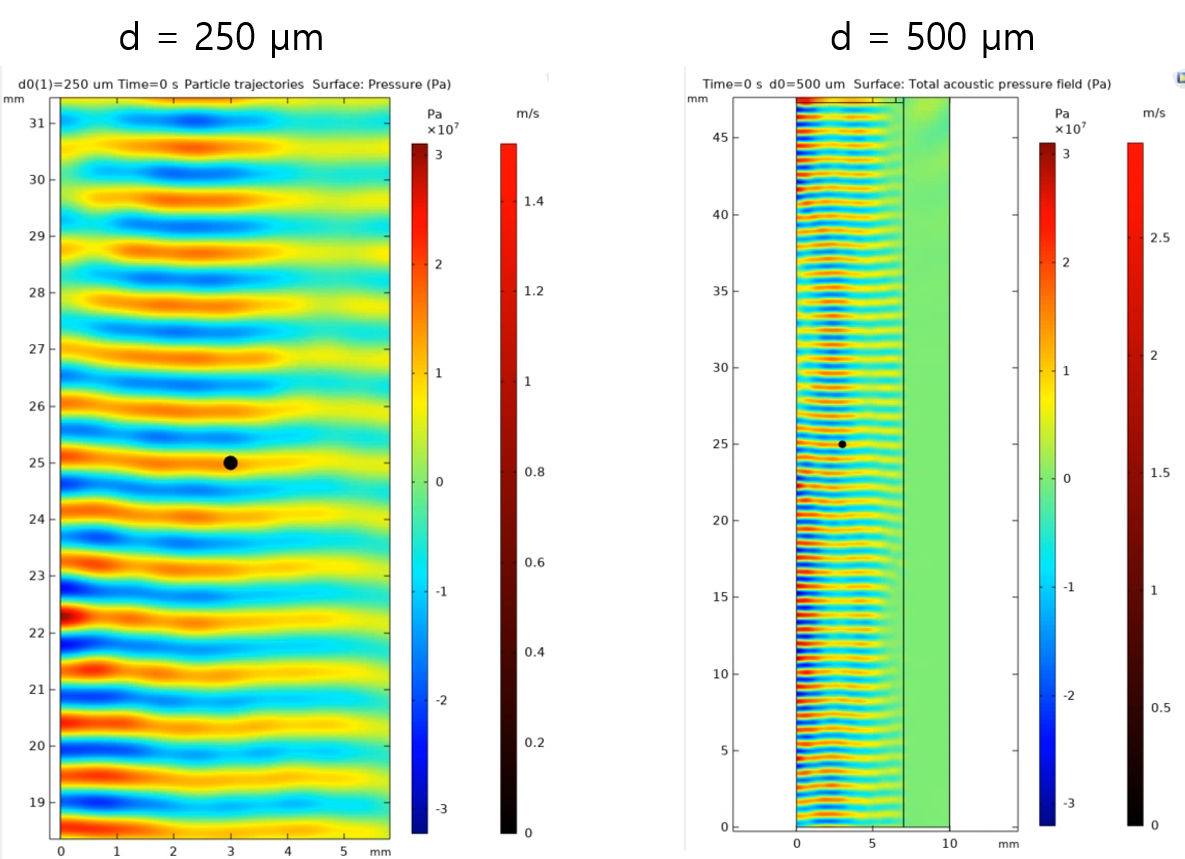


**Supplementary video 2.** The video clips for initial 0.1 s about the trajectories of two particles (250 and 500 μm in diameter) representing different sized spheroids under the given acoustic pressure field. The 250 μm particle slightly moves and then stays at a stable position, however the 500 μm particle never stay at any stable position for 0.1s.
